# Daily steps are associated with walking ability in hospitalized patients with sub-acute stroke

**DOI:** 10.1038/s41598-022-16416-8

**Published:** 2022-07-17

**Authors:** Hiroki Kubo, Masashi Kanai, Masafumi Nozoe, Asami Inamoto, Akira Taguchi, Kyoshi Mase, Shinichi Shimada

**Affiliations:** 1 Department of Rehabilitation, Itami Kousei Neurosurgical Hospital, 1-300-1, Nishino, Itami City, Hyogo Japan; 2 Department of Physical Therapy, Faculty of Nursing and Rehabilitation, Konan Women’s University, Kobe, Japan; 3 Department of Neurosurgery, Itami Kousei Neurosurgical Hospital, Itami, Japan

**Keywords:** Neuroscience, Health care, Neurology

## Abstract

Increased physical activity is required in patients with stroke that are hospitalized in the rehabilitation unit. This study investigated the association between the daily number of steps and walking independence in order to determine the cutoff value of daily number of steps that can predict walking independence in hospitalized patients with sub-acute stroke. This cross-sectional observational study included 85 stroke patients admitted to the rehabilitation unit. The average daily number of steps was measured using Fitbit One for 4 days starting at 30 days after stroke onset. 6-min walk test, and Fugl-Meyer assessment of the lower extremities were measured The category of walking independence was classified using the Functional Ambulation Category (FAC). The subjects were divided into two groups according to the FAC score: a walking independence group (FAC ≥ 4) and a walking non-independence group (FAC ≤ 3). Logistic regression analysis was conducted to investigate the association of daily number of steps with walking independence and a receiver operating characteristic curve was used to identify the cutoff value of daily number of steps for predicting walking independence. The daily number of steps (per 1000 steps) was independently associated with walking independence (odds ratio (OR); 2.53, 95% confidence interval (CI); 1.40–5.73, *p* = 0.009). The cutoff value of daily number of steps for predicting independent walking was 4286 steps (area under the curve = 0.914, sensitivity of 0.731, and specificity of 0.949). The daily number of steps was associated with independent walking in hospitalized patients with sub-acute stroke. The daily number of steps may be a useful target in rehabilitation for patients with sub-acute stroke.

## Introduction

Physical activity is important for promoting functional recovery and secondary stroke prevention^1^; however, a previous study found that insufficient physical activity was higher in individuals with stroke than those without stroke^2^. Moreover, most of the patient’s day was spent inactive (median 48.1%), alone (median 53.7%), and in their bedroom (median 56.5%) in hospitalized stroke patients^3^. In a rehabilitation unit, hospitalized sub-acute stroke patients took fewer steps than chronic home-living stroke patients^4^. Light intensity physical activity was associated with improved walking independence^5^ and physical activity time was associated with activities of daily living (ADL) score^6^ in rehabilitation unit stroke patients. Hence, increasing the physical activity might be required in patients with stroke hospitalized in the rehabilitation unit.

Improved walking ability is a rehabilitation goal for stroke patients hospitalized in the rehabilitation unit^7^. Previous studies showed that walking independence was positively associated with the amount of physical activity in patients with stroke hospitalized in the rehabilitation unit, however, they either have a small sample size or did not adjust fully^5,8^. Additionally, similar physical activity patterns were observed in moderate and severe stroke patients but was different in patients with mild stroke^8^. As intensity-based physical activity was non-linear due to walking ability in patients with stroke hospitalized in the rehabilitation unit^9^, there may be a threshold in step count which, if attained, is associated with the greatest increase in walking independence. Knowing these values of physical activity for predicting walking independence may be useful; however, to the best of our knowledge, this has not yet been investigated.

The objective of this study was to investigate the association of daily number of steps with walking independence and to identify the cutoff value of daily number of steps for predicting walking independence in patients with sub-acute stroke admitted to the rehabilitation unit.

## Methods

### Study design and subjects

**Figure 1:**
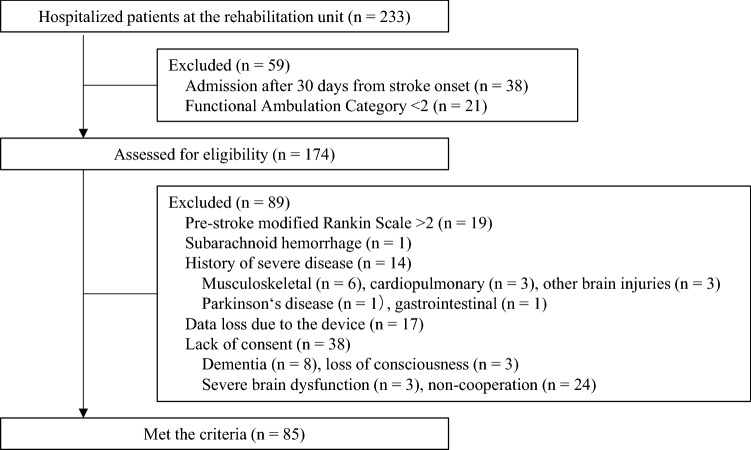
Participant flow in the present study.

In this cross-sectional study, the part of the data set from the previous study was used^10^, because the daily number of steps were measured from October 2017. Consecutive stroke patients were admitted to the rehabilitation unit at Itami Kousei Neurosurgical Hospital between October 2017 and September 2019. Eligibility criteria were those who were admitted to the rehabilitation unit within 30 days from stroke onset, could walk alone, or could walk with an intermittent or continuous light touch to assist balance or coordination (Functional Ambulation Category [FAC] ≥ 2). Exclusion criteria were as follows: pre-stroke modified Rankin Scale (mRS) score > 2; patients with subarachnoid hemorrhage; a history of severe diseases such as musculoskeletal disease, cardiopulmonary disease, other brain injuries, Parkinson’s disease, and gastrointestinal disease that the physician considered to have affected the ability to walk; or inability to provide consent due to loss of consciousness, aphasia, dementia, or non-cooperation (Fig. 1). All patients underwent 40–60 min sessions of supervised rehabilitation such as physical therapy, occupational therapy, and speech-language-hearing therapy 2–3 times a day, 7 times a week. This study was approved by the Konan Women’s University Research Committee (acknowledgment number: 2015020) and was in accordance with the Declaration of Helsinki. All participants provided informed consent. All research was performed in accordance with relevant guidelines or regulations.

### Patient demographics and clinical characteristics

Age, sex, height, weight, stroke type (cerebral infarction or intracerebral hemorrhage), disease history, and pre-stroke mRS were recorded upon admission and discharge from the rehabilitation unit. Body mass index (BMI) was calculated by dividing the body weight by the height squared (kg/m^2^). Stroke severity using the National Institutes of Health Stroke Scale (NIHSS) was assessed at 30 days from stroke onset.

### Outcome measurement

#### Physical activity

Physical activity was measured from thirty days after stroke onset. All patients wore the Fitbit One (Fitbit. Inc., San Francisco, CA, USA) on their non-paretic ankle^11,12^ for six days continuously except when bathing and sleeping. Fitbit One has good test–retest reliability across 3 days of free-living activity in stroke survivors^13^. Thus, the average daily number of steps taken during the middle 4-day period of the 6 days was used as the value of daily physical activity. Fitbit One is a three-dimensional accelerometer that calculates steps taken, floors climbed, distance traveled, and sleep quality. It has been used for stroke patients with demonstrated accuracy^12^. Patients were instructed to wear Fitbit One immediately after waking and remove it while bathing and before going to bed. They were instructed to continue with their activities of daily living without the need for excessive exercise, and they did not receive any feedback from the physical therapist. The number of steps was extracted by downloading the data files to Fitbit online dashboard software^14^. Each patient’s setup parameters, such as age, sex, height, and weight were fed in the device software to ascertain the outcome.

#### FAC

Patients were classified by category of walking independence using FAC^15^ at 30 days after stroke onset and discharge from the rehabilitation unit by a physical therapist. The FAC has excellent reliability, good concurrent and predictive validity, and good responsiveness in patients with hemiparesis after stroke^16^. It has six levels (0 to 5) that are classified according to the walking ability based on the amount of physical support required as follows: nonfunctional ambulatory (FAC 0); ambulatory [level II], continuous manual contact to support the body weight and maintain balance or to assist with coordination (FAC 1); ambulatory [level I], intermittent or continuous light touch to assist with balance or coordination (FAC 2); ambulatory, dependent on supervision (FAC 3); ambulatory, independent, level surface only (FAC 4); and ambulatory, independent (FAC 5).

#### Fugl-Meyer assessment of the lower extremities

Lower extremity motor impairment following stroke was assessed using the lower limb Fugl-Meyer assessment (FMA)^17^ at 30 days after stroke onset. The lower limb FMA score ranges from 0 (hemiplegia) to a maximum of 34 points. Seventeen items are included in the lower limb FMA and each item is scored on a 3-point ordinal scale (0 = cannot perform, 1 = performs partially, and 2 = performs fully). The lower limb FMA is best administered by a trained physical therapist.

#### Six-minute walk test

The 6MWT was used to evaluate the walking endurance at 30 days after stroke onset. A 30-m indoor walkway was used to conduct the test according to the ATS guidelines^18^. Patients were then instructed to walk from the starting line to the end of the walkway as many times as possible in 6 min. Patients were allowed to use their usual assistive devices and intermittent assistance for fall prevention was provided as necessary. During the test, patients were permitted to slow down, stop, and rest by leaning against the wall as necessary, but they resumed walking as soon as possible. Feedback was given and the distance covered at the end of the 6MWT was recorded.

### Statistical analysis

Descriptive statistics are presented as numbers (percentages) and median (interquartile range [IQR]) in all subjects. Shapiro–Wilk test was performed to check for normality. To investigate the difference between the daily number of steps and walking ability measured by FAC, the Kruskal–Wallis test was used when normality was not confirmed for continuous and ordinal variables following previous studies. When a statistically significant effect was found, the Steel–Dwass test was used as a multiple comparison to determine the difference between the daily number of steps and FAC in all subjects and by sex. Then, the subjects were divided into two groups according to the FAC score: a walking independence group (FAC ≥ 4) and a walking non-independence group (FAC ≤ 3). Logistic regression analysis was also performed to investigate the association of the daily number of steps (in increments of 1,000 steps) with walking independence adjusted by age, sex, stroke type (cerebral infarction or intracerebral hemorrhage), NIHSS, lower extremity FMA, and 6MWT following previous studies^19,20^. Multicollinearity was assessed using the variance inflation factor (VIF) and defined as VIF ≥ 10^21^. The receiver operating characteristic (ROC) curve analysis was conducted to establish the cutoff value of daily number of steps for predicting walking independence in all subjects. The area under the curve, sensitivity, and specificity were calculated to determine the optimal cutoff point. The optimal cutoff was determined using the Youden index, which was defined as the maximum vertical distance between the ROC curve and the diagonal or chance line, and was calculated as the maximum sum of sensitivity and specificity minus one^22^. Significance was set at < 0.05; all analyses were performed using JMP version 10.0 software (SAS Institute Japan, Tokyo, Japan).

## Results

**Figure 2:**
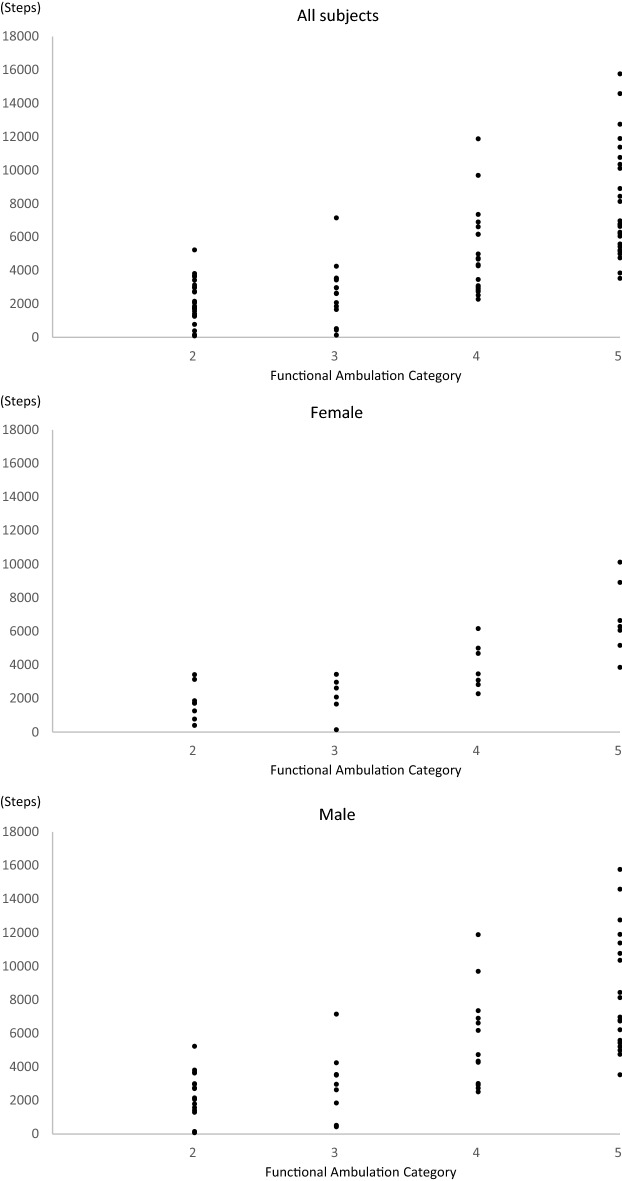
Scatter plot of Functional ambulation category and daily number of steps.

Patient demographics and clinical characteristics are presented in Table 1. A total of 233 patients with stroke were hospitalized during the study inclusion period. Of these patients, 148 were excluded from the study, and 85 met the inclusion criteria and were enrolled in the study. Normality of the daily number of steps could not be confirmed, the Kruskal–Wallis test and the Steel–Dwass test was used to investigate the differences among FAC. The median daily number of steps was 3,642 steps (IQR, 4,052 steps/day), 3,115.5 (IQR, 3,364.7 steps/day), and 4,286 (IQR, 4,213.5 steps/day) in all subjects, female and male, respectively (Table 2). There were significant interaction effects between the daily number of steps and FAC in all subjects (*p* < 0.001) (Table 2, Fig. 2).

**Table 1:** Patient demographics and clinical characteristics.

	All subjects n = 85
Age (years), (IQR)	69.0 (17.0)
Sex (female), n (%)	28 (32.9)
Height (cm), (IQR)	163.0 (14.0)
Weight (kg), (IQR)	62.4 (14.9)
BMI (kg/m^2^), (IQR)	23.4 (4.4)
**Type of stroke, n (%)**	
Cerebral infarction	52 (61.2)
Cardio-embolism	9 (10.6)
Large-artery atherosclerosis	15 (17.6)
Small-vessel occlusion	26 (30.6)
Unknown	2 (2.4)
Intra-cerebral hemorrhage	33 (38.8)
**Affected side, n (%)**	
Right	30 (35.3)
Left	53 (62.4)
Both	2 (2.3)
NIHSS (score), (IQR)	3.0 (5.0)
**History of diseases, n (%)**	
Hypertension	40 (52.9)
Diabetes mellitus	16 (18.8)
Cardiovascular disease	10 (11.8)
Pulmonary disease	4 (4.7)
Lipidemia	11 (12.9)
Cerebrovascular disease	17 (20.0)
Kidney disease	4 (4.7)
**Pre-stroke mRS**	
Score 0, n (%)	68 (80.0)
Score 1, n (%)	11 (12.9)
Score 2, n (%)	6 (7.1)
Lower limb FMA (score), (IQR)	32.0 (6.0)
6MWD (m), (IQR)	300.0 (274.7)

BMI, Body mass index; FAC, Functional ambulation category; FMA, Fugl-Meyer Assessment; IQR, inter quartile range.

**Table 2:** Daily number of steps in each ambulation category.

	All subjects	FAC 2	FAC 3	FAC 4	FAC 5	*p*-value
**All subjects**						
Median (IQR)	3642 (4,052)	1970 (1770.5)	2646 (1,852)	4518.5 (3559.5)^a, b^	6763.5 (5082.2)^a, b, c^	< 0.001
**Female**						
Median (IQR)	3115.5 (3364.7)	1723 (1920.2)	2354.5 (1800.4)	3471 (2,169)	6295 (3747)^a, b^	< 0.001
**Male**						
Median (IQR)	4286 (4213.5)	2441 (2026)	2978 (2,714)	4742 (4,159.5)^a^	6978 (5950)^a, b^	< 0.001

FAC; Functional Ambulation Category, IQR; interquartile range.

The Kruskal–Wallis test was used to investigate the differences among FAC.

The significance between each FAC by the Steel–Dwass test are shown as follows;

^a^significant difference vs. FAC 2 *p* < 0.05.

^b^significant difference vs. FAC 3 *p* < 0.05.

^c^significant difference vs. FAC 4 *p* < 0.05.

The logistic regression analysis demonstrated that walking independence was only associated with the daily number of steps (odds ratio, 2.53; 95% confidence interval, 1.40–5.73; *p* = 0.009) (Table 3).

**Table 3:** Logistic regression analyses of association between average daily step count and walking independence.

	Unadjusted model Odds ratio (95% CI)	*p*-value	Adjusted model Odds ratio (95% CI)	*p*-value
Age	0.97 (0.93–1.01)	0.087	0.95 (0.88–1.03)	0.221
Sex (female)	0.78 (0.31–1.94)	0.594	1.94 (0.42–10.13)	0.405
Stroke type (intracerebral hemorrhage)	0.57 (0.23–0.14)	0.202	0.80 (0.17–3.76)	0.776
National Institutes of Health Stroke Scale	0.56 (0.42–0.72)	< 0.001	0.71 (0.45–1.06)	0.112
Lower-limb Fugl-Meyer assessment	1.30 (1.15–1.53)	< 0.001	0.89 (0.71–1.10)	0.275
6-min walk test	1.01 (1.01–1.02)	< 0.001	1.01 (0.99–1.02)	0.077
Daily number of steps (per 1,000 steps)	2.95 (1.95–5.16)	< 0.001	2.53 (1.40–5.73)	0.009

**Figure 3:**
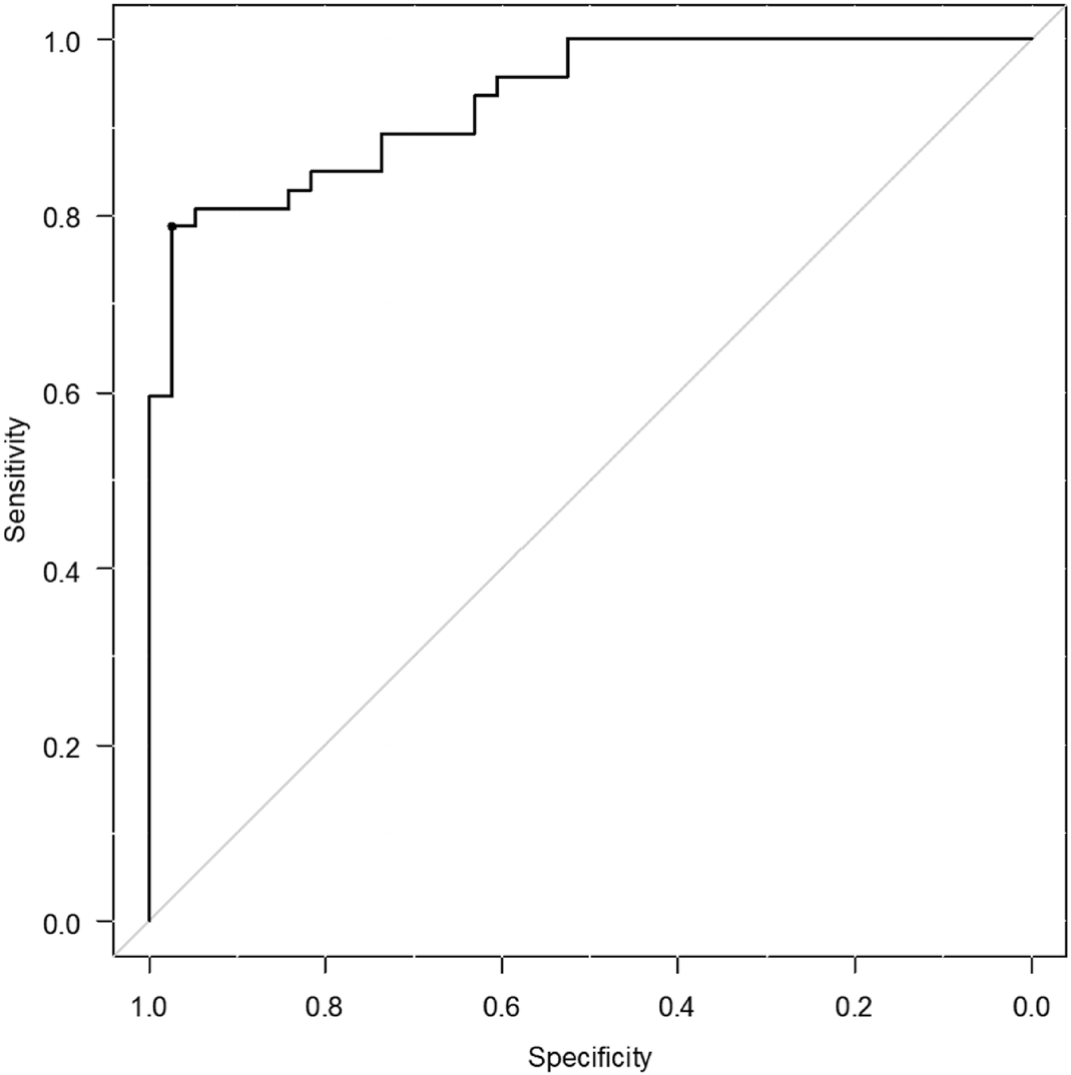
The cutoff value of the daily number of steps for determining walking independence. Cutoff value was 4286 steps (area under the curve = 0.914, sensitivity of 0.731, and specificity of 0.949).

Based on the ROC curve, the cutoff value of daily number of steps for predicting walking independence was 4286 steps (*p* < 0.005, area under the curve = 0.914, sensitivity of 0.731, and specificity of 0.949) (Fig. 3).

## Discussion

We demonstrated that the daily number of steps was associated with walking independence after adjusting for confounding factors such as age, sex, stroke type, NIHSS, lower extremity FMA, and 6MWT. Previous studies found that increasing day-time physical activity was related to walking independence in patients with sub-acute stroke who could not walk alone^5^, and gait independence increased the median percentage of time spent standing/walking^8^ by stroke patients in the rehabilitation unit, which partially support our results. Further studies with longitudinal designs are required to clarify the causal relationship between the daily number of steps and walking independence in stroke patients.

The cutoff value of the daily number of steps for predicting walking independence was 4,286 steps in patients with sub-acute stroke hospitalized in the rehabilitation unit. To our knowledge, the cutoff value of the number of steps for predicting walking independence has not been investigated. In a previous study, the cutoff value of 6MWT for determining walking independence was 304 m^10^ and the cutoff value of lower extremity FMA for predicting walking independence was 22.5 points^23^. As improving the walking ability is one of the goals of rehabilitation^7^, combining the cutoff value of the number of steps in our results with that of other motor performance tests may be useful to facilitate early walking independence. The results of this study suggest that for every 1,000 steps per day increase in the patient’s step count, the odds of becoming an independent ambulator increased between 1.398 and 5.734. Meanwhile, the sensitivity and specificity were 0.731 and 0.949, respectively in the present study; there were non-independent walking patients who achieved 4286 steps. Thus, for non-independent patients who achieved 4286 steps, it may be necessary to consider other factors, such as cognitive function^24^.

The daily number of steps was 4935 to 5291 in patients with mild stroke (NIHSS score 1 to 2)^25^ and 1514.3 to 3010.7 in patients with moderate stroke (FAC 3; range 2–5)^4^. Day-time physical activity differed according to severity measured by NIHSS in patients with acute stroke^26^. These results are partly consistent with our study findings that the number of steps decreases with increasing disability after stroke.

The number of steps in the walking independence group was 4518.5 steps/day (IQR, 3559.5 steps/day) for FAC 4 and 6763.5 steps/day (IQR, 5082.2 steps/day) for FAC 5. It was previously reported that 6025 steps per day is an initial target for reducing new vascular events after mild ischemic stroke^27^. The number of steps per day after discharge from the rehabilitation unit increased by 724 steps compared with during hospitalization in stroke patients^28^. In stroke patients with independent walking, preventing new vascular events is an important therapeutic target^27,29^. However, FAC 4 stroke patients did not reach the target level for reducing new vascular events. For these patients, improved walking ability for outdoor walking independence and promotion of physical activity may be required during hospitalization. In addition to physical function, environmental factors are involved in physical activity in community-dwelling stroke patients^30^. Thus, the factors associated with physical activity during hospitalization and after discharge may differ. Even patients with mild stroke who are able to walk outdoors independently may need consider environmental factors after discharge to maintain physical activity.

On the other hand, the number of steps in the walking non-independence group was 1,970 steps/day (IQR, 1770.5 steps/day) for FAC 2 and 2646 steps/day (IQR, 1852 steps/day) for FAC 3. Klassen et al. reported that the number of steps during a physical therapy session was 580 (SD, 440) for usual care physical therapy, 2169 (SD, 1106) for more than double the intensity of the control, and 4747 (SD, 2083) for more than quadruple the intensity of the control, and walking recovery was improved in the higher dose group^31^. Patients with FAC 2.3 in our study did not reach the values of the previous study; however there is still a possibility of improvement. In addition, daytime and non-therapy time light intensity physical activity were related to functional recovery in patients with sub-acute stroke who could not walk independently^5^. Thus improvement of the walking ability in patients with poor walking ability and increasing the number of steps during therapy time and light intensity physical activity during non-therapy time are important methods for recovering the walking ability.

### Study limitations

There were several limitations in the current study. First, the daily number of steps taken was used as the measure of physical activity; however, the Fitbit One mean error was higher for slower velocities (10.9% for speeds < 0.4 m/s)^12^. Thus, there may have been measurement errors of physical activity in stroke patients with low walking ability. Second, the daily number of steps was measured at 30 days after stroke onset in patients who were admitted to the rehabilitation unit. Thus, these results may only be applicable to stroke patients in the rehabilitation unit and not to acute stroke or community-dwelling chronic stroke patients. In addition, we could not measure long-term outcomes such as mRS or FAC at 3 or 6 months; thus the relationship between the number of steps at 30 days from stroke onset and long-term outcome was not clarified. Further studies are needed to investigate the step count associated with improvement in function during rehabilitation or beyond. Third, this was a cross-sectional study, and the causal relationship between the daily number of steps and walking independence was unknown. Additionally, the association between walking independence and age and severity also disappeared, which may possibly indicate a high correlation between the daily number of steps and age or stroke severity. Thus, further longitudinal studies with a larger sample size are required to clarify the causal relationship between the daily number of steps and walking independence. Fourth, although pre-stroke physical activity was associated with stroke severity and post-stroke outcome^32,33^, pre-stroke physical activity or fitness was not measured. Additionally, rehabilitation content affects physical activity; however, the contents of rehabilitation are not standardized because they are determined by each physician. Thus, these factors may affect the result of our study. Fifth, the wearing time of Fitbit One may vary depending on the patient’s lifestyle. Further studies are needed to consider wearing time to measure physical activity. Finally, this study did not use randomization or assignment; thus, there was a possibility of selection bias and lack of internal validity. Further studies with comparative validity designs are required.

## Conclusion

This study demonstrated the association between the daily number of steps and walking ability, and calculated the cutoff value for walking independence in patients with sub-acute stroke hospitalized in the rehabilitation unit. A daily number of steps of 4286 steps/day may be useful for determining walking independence in patients with sub-acute stroke.

## Supplementary Information

Supplementary Information.
