# Author Correction: Daily steps are associated with walking ability in hospitalized patients with sub-acute stroke

**DOI:** 10.1038/s41598-022-18802-8

**Published:** 2022-08-23

**Authors:** Hiroki Kubo, Masashi Kanai, Masafumi Nozoe, Asami Inamoto, Akira Taguchi, Kyoshi Mase, Shinichi Shimada

**Affiliations:** 1 Department of Rehabilitation, Itami Kousei Neurosurgical Hospital, 1‑300‑1, Nishino, Itami City, Hyogo Japan; 2 Department of Physical Therapy, Faculty of Nursing and Rehabilitation, Konan Women’s University, Kobe, Japan; 3 Department of Neurosurgery, Itami Kousei Neurosurgical Hospital, Itami, Japan

Correction to: *Scientific Reports* 10.1038/s41598-022-16416-8, published online 17 July 2022

The original version of this Article contained errors. After publication it transpired that the Supplementary Information file which accompanies the Article, inadvertently contained confidential patient data.

The error has now been corrected and this patient data has been removed in the Supplementary Information file.

